# Quality controls

**DOI:** 10.1186/s12915-015-0170-0

**Published:** 2015-08-06

**Authors:** Emma Saxon

**Affiliations:** BMC Biology, BioMed Central, 236 Gray’s Inn Road, London, WC1X 8HB UK

## Abstract

Some steroid drugs are designed for clinical use to combat muscle-wasting diseases, but are also used by athletes to improve their performance by increasing muscle strength or endurance. This study investigated the effect of one steroid drug on the number of times a weight could be lifted by healthy male subjects as a measure of endurance. The ratio of number of lifts performed with a steroid-injected arm versus a sham-injected control arm increased with the drug dose; the authors therefore concluded that the drug improved endurance.

## Commentary

**Fig. 1:**
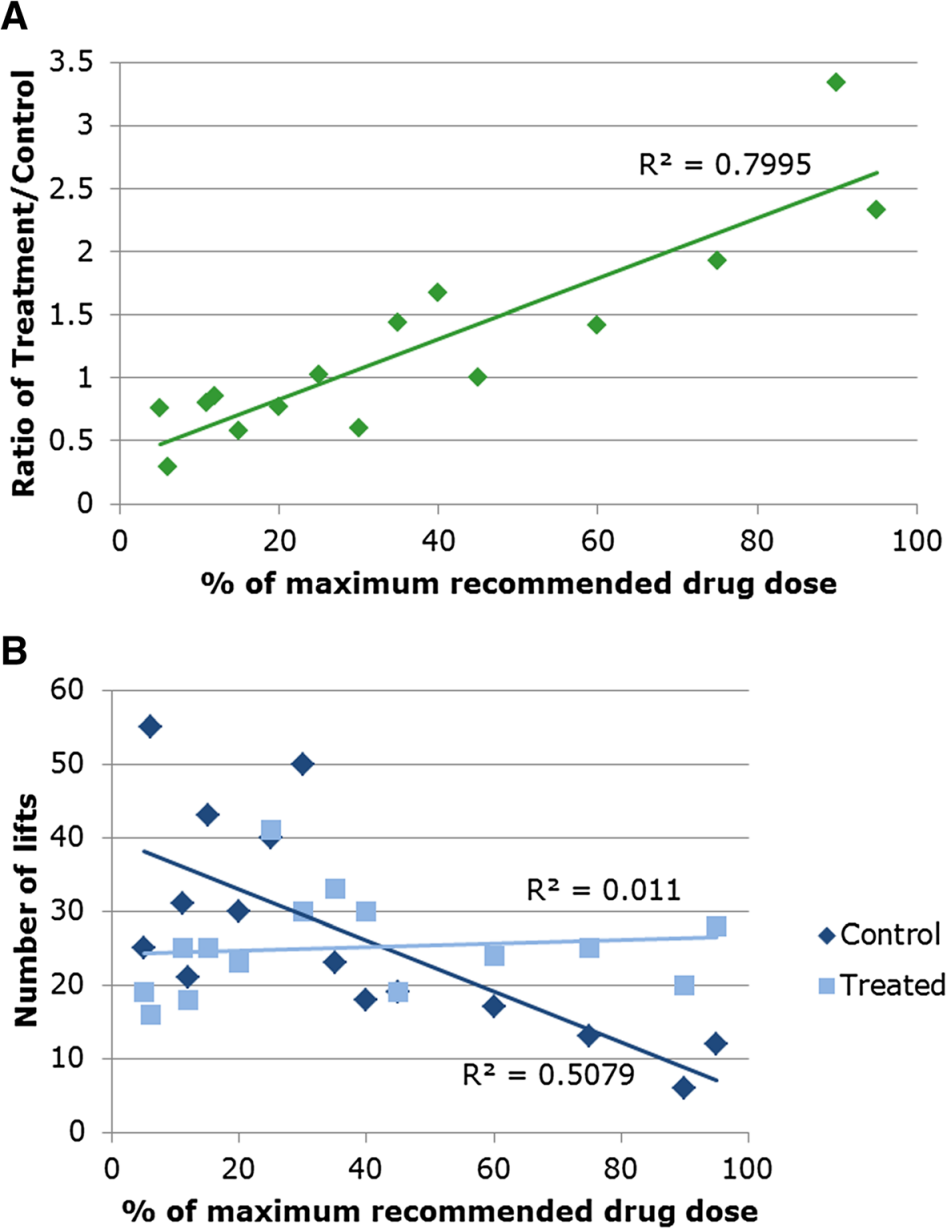
Number of weight lifts performed by healthy males in a steroid-injected arm compared with the control. **a** The ratio of lifts performed by steroid versus control arms across a range of drug doses. **b** The absolute number of lifts. R^2^ = regression analysis coefficient

Processing data for graphical display can sometimes hide underlying problems with the data. In this example, it seems that injecting a dose of performance-enhancing drugs into one arm of weightlifters significantly increases the number of repetitions they can perform before the onset of muscle fatigue, compared with the number achieved by the sham-injected control arm (Fig. 1a; regression analysis coefficient R^2^ = 0.7995, *p* < 0.001). The greater the proportion of the recommended safe dose of drugs injected, the higher the fold difference in lifting endurance compared with the control. But these processed data hide a somewhat surprising result, which was not clearly described in the study: lifting ability actually decreased in control arms with an increasing dose of the drug in experimental arms (Fig. 1b).

The control varies between treatments, and so the experimental to control ratio increases with drug level in the experimental arm largely, though not entirely, because the function of the control arm is worse. Regardless of whether this represents a real biological effect, the way the data were processed and displayed in the top graph was inappropriate — this example highlights the importance of examining raw and not just processed data.

